# The Yin and Yang of the Natural Product Triptolide and Its Interactions with XPB, an Essential Protein for Gene Expression and DNA Repair

**DOI:** 10.3390/genes15101287

**Published:** 2024-09-30

**Authors:** David Gorrie, Marco Bravo, Li Fan

**Affiliations:** Department of Biochemistry, University of California, 900 University Ave, Riverside, CA 92521, USA; david.gorrie@email.ucr.edu (D.G.); marco.bravo@email.ucr.edu (M.B.)

**Keywords:** anticancer drug, hepatotoxicity, inflammation, autoimmune disease, transcription

## Abstract

Triptolide, a bioactive diterpene tri-epoxide extracted from *Tripterygium wilfordii* Hook F (TWHF), exhibits notable pharmacological activities, including anti-inflammatory, immunosuppressive, antifertility, and anticancer effects. Despite its promising therapeutic potential, clinical applications of triptolide are significantly limited by its poor water solubility and substantial toxicity, particularly hepatotoxicity, nephrotoxicity, and cardiotoxicity. These toxic effects are difficult to separate from many of its desired therapeutic effects, the Yin and Yang of triptolide applications. Triptolide’s therapeutic and toxic effects are linked to its inhibitory interactions with XPB, a DNA helicase essential for transcription by RNA polymerase II (RNAPII) and nucleotide excision repair (NER). By irreversibly binding to XPB, triptolide inhibits its ATPase activity, leading to global repression of transcription and impaired NER, which underlies its cytotoxic and antitumor properties. Recent developments, including triptolide prodrugs such as Minnelide and derivatives like glutriptolides, aim to enhance its pharmacokinetic properties and reduce toxicity. This review critically examines triptolide’s chemical structure, therapeutic applications, toxicological profile, and molecular interactions with XPB and other protein targets to inform future strategies that maximize therapeutic efficacy while minimizing adverse effects.

## 1. Introduction

Triptolide is a natural product derived from the Chinese medicinal plant TWHF [1]. TWHF, with the common Chinese name Lei Gong Teng (meaning thunder god vine), was traditionally used to treat rheumatoid arthritis, systemic lupus erythematosus, nephritis, cancer, and psoriasis in Chinese medicine for centuries [2,3]. Since its first identification and isolation from TWHF in 1972 [4], triptolide has been recognized as the primary functional component of TWHF and gained significant attention for its diverse biological activities, such as anti-inflammatory, immunosuppressive, anti-neoplastic, and antitumoral activities [5,6,7]. In fact, according to clinicaltrials.gov (a website maintained by the US National Institutes of Health), five clinical trials are currently investigating triptolide derivatives for the treatment of various cancers [8]. Triptolide was also reported to exist in *T. hypoglaucum* (Levl*)* Hukeda, *T. forretii* Dials, and *T. regelli* Sprague et Taketa [1].

Triptolide has significant impacts on cancer cell survival and proliferation. In the NCI-60 screen, triptolide showed activity against all lines with IC50 values between 2.6 and 103 nM [7,9]. Numerous studies have confirmed triptolide to induce apoptosis and inhibit the growth and metastasis of a broad range of human tumor cells [10,11,12,13,14]. Inhibition of NER and general transcription are both consequences of triptolide binding to XPB, mediating cell death in cancer cells after triptolide treatment [5,15,16]. XPB is a DNA-dependent ATPase, or helicase. It is a subunit of TFIIH, indispensable for transcription initiation and NER [17]. A result of triptolide/XPB binding is a global inhibition of transcription accompanying the arrest of TFIIH on promoters, a proposed mechanism for triptolide [9,15]. Apoptosis is triggered by the reduction in antiapoptotic proteins such as XIAP and Bcl-2 in some cell types [10,18]. Transcriptional repression of Mcl-1 has also been shown to trigger apoptosis in myeloma [19].

The interaction of triptolide with XPB also leads to the inhibition of NER, providing a rationale for triptolide as an adjuvant to standard chemotherapies currently in the clinic. For platinum-based chemotherapies, when co-administered with carboplatin in melanoma lines, triptolide was shown to enhance the effect of the platin by inhibiting NER [11]. When combined with cisplatin in lung cancer lines, triptolide exhibited a synergistic effect by inhibiting the NER pathway [12]. In another study, minnelide, a triptolide prodrug, inhibited NER, overcoming oxaliplatin resistance in pancreatic cancer models [20].

Triptolide has displayed tremendous potential medical benefits in treating various diseases by manipulating many signal pathways essential for inflammation, immune responses, cell death, proliferation, and even global inhibition of genome transcription. Unfortunately, its diverse biological activities are also the cause for systemic toxicity involving multiple organs and tissues, the Yin and Yang of triptolide applications. In this review, we focus on the chemical structure and properties of triptolide and the molecular mechanisms of its biological actions. We aim to inspire new strategies to improve the efficacy of future triptolide applications with reduced or no toxicity.

## 2. Chemical Properties of Triptolide and Existing Structure–Activity Relationship (SAR) Studies

**Figure 1:**
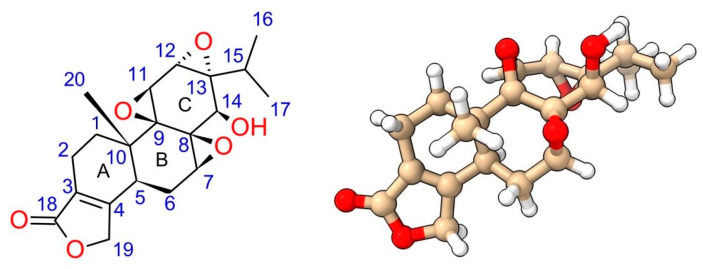
Structure of triptolide. The oxygen atoms and hydroxide group are highlighted in red.

Triptolide, also named PG490 or LLDT-2, is a diterpenoid tri-epoxide (C_20_H_24_O_6_, molecular weight: 360.4) (Figure 1). Structure–activity relationship (SAR) studies have elucidated several essential structural moieties responsible for its bioactivity. Critical moieties include the C-14-hydroxyl, 12,13-epoxide, 9,11-epoxide, and 7,8-epoxide, along with the butenolide and C-5,6 positions. Understanding these structural components is essential for improving triptolide’s therapeutic potential. Each of these moieties contributes uniquely to the molecule’s overall activity.

The 12,13-epoxide is particularly vital for triptolide’s biological activity, contributing to its interaction with cysteine residues on the XPB protein, a component of the TFIIH transcription factor complex [16,21]. This interaction is crucial for triptolide’s mechanism of action. C-12 is the hot spot for nucleophile attack. Interestingly, ring-opening at C-12 led to the loss of its immunosuppressive activity but with limited impact on its anti-inflammation activity [22].

Modifications at the 9,11-epoxide have shown some flexibility without complete loss of activity, although retaining this epoxide is generally preferable for maintaining bioactivity [23]. The ability to change this site without entirely losing efficacy suggests some structural tolerance, providing opportunities for drug development. However, these modifications must be carefully designed to avoid compromising triptolide’s therapeutic effects.

The 7,8-epoxide is essential; ring-opening modifications at this site significantly reduce biological efficacy, underscoring its critical role in maintaining the molecule’s biological activity [1,23]. Studies showing its alteration can drastically diminish triptolide’s effectiveness highlight its importance. Maintaining this structural feature is thus paramount for preserving the drug’s bioactivity. Researchers must ensure that any derivative keeps the integrity of the 7,8-epoxide to avoid reducing its therapeutic potential.

The butenolide moiety, particularly the C-18 carbonyl group, is crucial for binding to target proteins, including XPB [23]. Structural studies and synthetic analogs have demonstrated that the butenolide ring, when modified, can drastically alter cytotoxicity and biological function, with certain analogs retaining activity only when the five-membered lactone ring remains intact [23]. These structural studies underscore the significance of the butenolide moiety in triptolide’s bioactivity. Ensuring this moiety’s preservation in synthetic derivatives is vital for keeping their efficacy.

**Figure 2:**
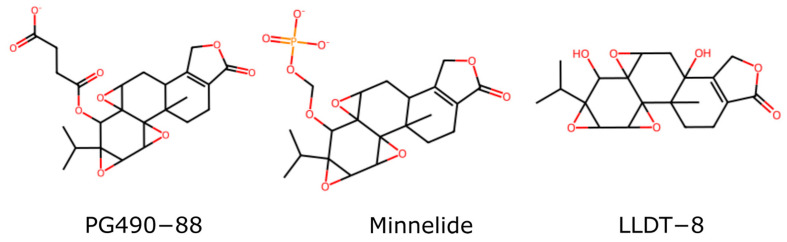
Three water-soluble triptolide derivatives.

Modifications to the C-14-hydroxyl have been extensively explored to improve water solubility and reduce toxicity. Prodrugs such as PG490-88 (or F60008) and Minnelide (Figure 2) are examples where the C-14 hydroxyl is changed to enhance pharmacokinetic properties, resulting in a prodrug metabolized to triptolide in vivo. Interestingly, some modifications at the C-14 position that do not result in prodrugs still retain significant biological activity, challenging earlier assumptions about the necessity of the C-14 hydroxyl group for activity [23,24]. These findings open new avenues for drug design, allowing for greater flexibility in changing this position.

Finally, modifications at the C-5,6 positions have revealed that the trans-A/B ring junction is critical for maintaining cytotoxicity. Studies suggest that the correct stereochemistry at these positions is necessary for the best biological activity [23]. Any deviation from this configuration may lead to a significant reduction in efficacy.

## 3. Potential Medical Applications

**Figure 3:**
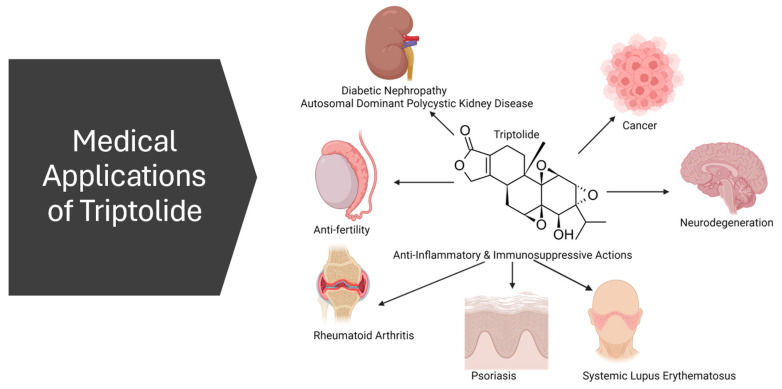
Schematic summary of triptolide potential medical applications.

Triptolide has emerged as a versatile candidate in medical research, showcasing significant anti-inflammatory, immunosuppressive, and anti-neoplastic properties [5]. Its broad spectrum of biological activities makes it a compound of interest for various therapeutic applications (Figure 3).

### 3.1. Anti-Inflammatory and Immunosuppression

Triptolide’s anti-inflammatory effects are mediated by suppressing NF-κB at the transcription level by inhibiting RNAPII. NF-κB is a family of transcription factors key to immune function and inflammation. By inhibiting this transcription factor, triptolide decreases a multitude of inflammatory mediators such as Tumor Necrosis Factor (TNF)-α [25]. By reducing circulating levels of this factor, triptolide achieves an anti-inflammatory effect [26].

In immunosuppression, triptolide’s ability to downregulate immune responses holds promise for applications in organ transplantation and autoimmune disorders like lupus. Again, its effects on transcription, involving RNAPII and NF-κB, contribute to its immunosuppressive actions. Also, by inhibiting NF-κB, triptolide inhibits cellular immunity. Triptolide both inhibits T cell function and activation. It inhibits the expression of interleukin-2 and cytokine genes in T cells [1,27]. These actions have made triptolide a potentially helpful treatment for inflammatory and autoimmune diseases.

In summary, triptolide has been used to treat rheumatoid arthritis, systemic lupus erythematosus, lupus nephritis, inflammatory bowel disease, colitis, and asthma due to its potent anti-inflammatory and immunosuppressive actions [28]. These effects can be attributed in large part to triptolide’s effects on the NF-κB transcription factor [29].

### 3.2. Anticancer

Triptolide’s anticancer potential is evident through its apoptosis-inducing effects on various cancer cell lines. Its impact on transcription, via its binding to XPB and the subsequent effects on RNAPII, results in decreased antiapoptotic proteins such as XIAP and Bcl-2 and specific miRNAs, inducing apoptosis [10,18,30]. Additionally, its inhibition of NER enhances the effectiveness of platinum-based chemotherapies. Though not as impactful as the global suppression of transcription, the consequences of triptolide’s inhibition of NER are significant, particularly in overcoming platinum-based chemotherapy resistance and synergism with administered platin therapy. By preventing NER, triptolide promotes the accumulation of DNA damage, triggering apoptosis in cancer cells [11,12,20]. Not only does triptolide act synergistically with drugs like cisplatin and carboplatin, in combination with medications such as Idarubicin or Ara C, it has also been found to exhibit a synergistic effect that triggers apoptosis in THP-1 leukemic cells and primary blast cells derived from a patient with acute myeloid leukemia (AML) [31,32].

In summary, triptolide has shown promise against lung, liver, pancreatic, prostate, and other solid tumors and synergism with many chemotherapies currently in the clinic. Triptolide accomplishes this by binding to XPB and inhibiting transcription and NER to promote apoptosis and autophagy [28]. NF-κB is again highly implicated [29]. However, in the context of cancer, numerous other factors (mRNA, proteins, miRNA, etc.) impacted by the general inhibition of transcription are also involved [28]. Interestingly, research has shown that triptolide induces apoptosis in cancer cells through both p53-dependent and p53-independent mechanisms. In cancer cells where p53 is functional, triptolide actually enhances p53 activity by increasing protein levels of p53 in many cancer cell lines, leading to an increase in the expression of pro-apoptotic proteins such as Bax, PUMA, and p21, which promotes cell cycle arrest and apoptosis [18,33,34,35]. However, the relationship between triptolide and p53 is nuanced. While triptolide can elevate p53 levels, it does not always lead to the activation of all p53 target genes. For instance, in certain contexts, triptolide may inhibit the transcriptional activity of p53, affecting its ability to regulate specific target genes [34].

### 3.3. Antifertility

Triptolide can exert a sterilizing effect on males that is at least partially reversible. This has led to interest in the development of triptolide as a male contraceptive [36]. LLDT8 (Figure 2) is one example of a triptolide derivative that completed clinical trials and was tolerated well [37]. Although it was initially developed as a powerful anti-inflammatory and immunosuppressive drug, it has also been shown to cause a block in meiosis in the testes of mice and rats. This results in sperm abnormalities and drug-induced sterility [38,39]. This effect is attributable to XPB binding and the inhibition of transcription in the testes.

Triptolide has been extensively studied for its antifertility effects in male mammals. Research highlights its potential as a male contraceptive, noting both its efficacy and the mechanisms by which it impairs fertility. Studies consistently show that triptolide exerts significant effects on sperm motility and morphology, leading to infertility without severely impacting overall testicular function or hormone levels.

A notable study by Sinha Hikim et al. (2013) demonstrated that triptolide induces infertility in male rats primarily by impairing the ultrastructure of cauda epididymal sperm [40]. Oral administration of triptolide at 100 µg/kg body weight daily for 70 days led to complete infertility, with little effect on spermatogenesis and Leydig cell function. Despite normal testicular histology and hormone levels, epididymal sperm showed severe defects, including the absence of plasma membrane over the tail, nuclear decondensation, and disorganized mitochondrial sheath. These findings suggest that the compound’s antifertility effects are primarily post-testicular, targeting sperm maturation and function in the epididymis [40].

Lue et al. (2013) explored triptolide’s potential as a male contraceptive, confirming its effectiveness at doses that induce infertility without significant adverse effects on the testes [41]. In their study, male rats administered triptolide displayed drastically reduced sperm motility and cauda epididymal sperm content yet maintained normal levels of luteinizing hormone (LH), follicle-stimulating hormone (FSH), and testosterone. This indicates that triptolide’s primary action occurs after spermatogenesis, affecting sperm post-testicular maturation and function. The study also noted modest decreases in seminiferous tubule volume and round spermatid numbers but no significant changes in overall testicular morphology or function, reinforcing the notion of a post-testicular site of action [41].

In another study, researchers investigated the molecular mechanisms underlying triptolide-induced testicular toxicity using a combination of metabolomics, cytotoxicity assays, and molecular docking. The study revealed that triptolide disrupts vital metabolic pathways and induces cytotoxic effects in testicular cells. Metabolomic analysis showed alterations in metabolites related to energy metabolism, amino acid metabolism, and oxidative stress, suggesting that triptolide induces metabolic stress and oxidative damage in testicular tissues. These findings provide insights into the cellular and molecular pathways affected by triptolide, contributing to its antifertility effects [42].

A study by Xiong (2019) highlighted the apoptotic effects of triptolide on male germ cells and its epigenetic impact [43]. Triptolide was found to induce apoptosis in germ cells, disrupting spermatogenesis and leading to reduced sperm count and quality. Additionally, the study observed significant epigenetic modifications, including altered histone acetylation patterns, which may contribute to the long-term effects of triptolide on male fertility. These epigenetic changes underscore the compound’s potential to cause lasting damage to the reproductive system [43]. These studies show that there are still significant safety concerns about the use of triptolide as a male contraceptive.

Studies focusing on fertility recovery post-treatment indicate that the compound’s effects are reversible upon cessation of administration. Male rats treated with triptolide for a limited period regained fertility within a few months after stopping the treatment, suggesting that the antifertility effects of triptolide do not cause permanent damage to the reproductive system. This reversibility makes triptolide an attractive candidate for developing a reversible male contraceptive [44]. At least at some dosages, there is recovery of at least some fertility post triptolide exposure.

The cumulative research on triptolide underscores its potential as a male contraceptive by elucidating its mechanisms of action and highlighting its reversible and non-permanent effects on fertility. The compound’s ability to impair sperm function post-spermatogenesis, coupled with its minimal impact on hormonal levels and testicular structure, positions it as a promising candidate for male contraception. However, further studies are needed to fully understand the long-term effects, optimal dosing, and safety profiles to advance its application in human contraception.

### 3.4. Nephropathy

Triptolide has garnered significant attention for its therapeutic potential in various diseases, including diabetic nephropathy (DN) and autosomal dominant polycystic kidney disease (ADPKD). ADPKD is a chronic kidney disorder characterized by the formation of numerous cysts within the kidneys, leading to progressive renal failure. This disease is primarily caused by mutations in either the PKD1 or PKD2 genes, which encode the proteins polycystin-1 (PC-1) and polycystin-2 (PC-2), respectively [45,46,47,48].

The pathogenesis of ADPKD involves multiple mechanisms, including abnormal epithelial cell proliferation, fluid secretion, and extracellular matrix remodeling. Polycystins play a critical role in these processes, as they are involved in mechanosensation and calcium signaling within the primary cilia of renal epithelial cells [46]. Mutations in PKD1 or PKD2 disrupt these signaling pathways, leading to uncontrolled cell proliferation and cyst formation. Specifically, the loss of function in these genes impairs the release of calcium in response to ciliary bending, which is a key signal for maintaining epithelial cell quiescence [45].

Emerging evidence suggests that triptolide can mitigate the progression of ADPKD by targeting these underlying mechanisms. Triptolide has been shown to induce calcium release through a PC-2-dependent pathway, effectively arresting the growth of cystic cells [45]. In a study by Leuenroth et al. (2008), triptolide treatment significantly reduced cyst formation and growth in a murine model of ADPKD [46]. This effect was attributed to the upregulation of p21, a cyclin-dependent kinase inhibitor, which led to the arrest of cell cycle progression in PKD1-deficient cells [45].

Further research by the same group proved that triptolide’s therapeutic efficacy extends beyond its antiproliferative effects. Triptolide also modulates the NF-κB signaling pathway, which is implicated in inflammation and cystogenesis in ADPKD. By inhibiting NF-κB transactivation, triptolide reduces the expression of pro-inflammatory cytokines and chemokines, thereby attenuating the inflammatory response which contributes to cyst expansion and renal damage [46].

In addition to its effects on cell proliferation and inflammation, triptolide influences other cellular pathways relevant to ADPKD pathogenesis. Triptolide has been shown to inhibit the mammalian target of rapamycin (mTOR) pathway, which is involved in cell growth, proliferation, and survival. The mTOR pathway is abnormally activated in ADPKD, promoting cyst growth and disease progression. Inhibition of this pathway by triptolide further supports its potential as a multifaceted therapeutic agent for ADPKD [45,48].

### 3.5. Neurodegeneration

While less explored than some of triptolide’s other applications, triptolide has shown usefulness in treating neurodegenerative diseases. Triptolide, a compound not initially known for its neuroprotective properties, has been found to shield nerve cells in inflammation conditions. Studies have shown that it can inhibit the production of TNF-α, a particularly beneficial process in the context of Alzheimer’s Disease (AD) [49]. Additionally, triptolide has been observed to stimulate the production of neurotrophic factors, thereby offering further protection to neurons [50]. In a separate study involving an AD mouse model, treatment with triptolide was found to improve learning and memory deficits and reduce the buildup of β-amyloid protein in the brain [51].

## 4. Toxicity

**Figure 4:**
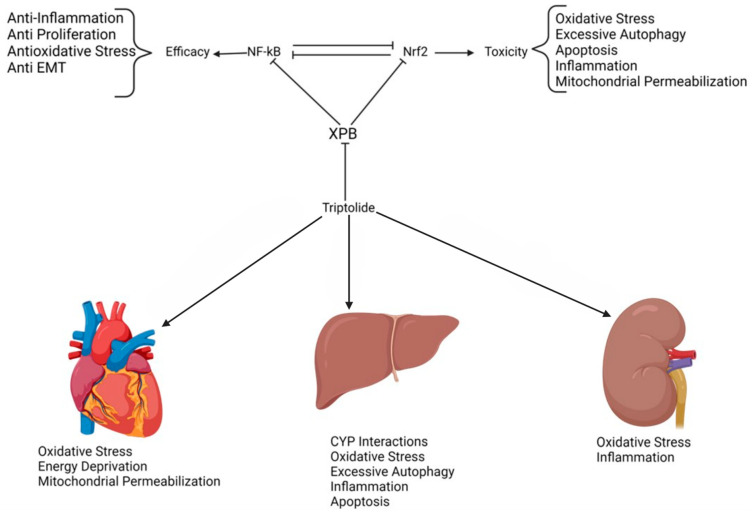
Mechanisms of triptolide toxicity.

The clinical application of triptolide is significantly hindered by its pronounced toxicity, particularly hepatotoxicity. The primary mechanism underlying triptolide’s toxicity is its broad-spectrum inhibition of transcription, affecting key transcription factors such as NF-κB, Nrf2, p53, and HSP70 (Figure 4). Toxicological studies have shown that triptolide doses of 1.2 mg/kg and 2.4 mg/kg cause significant tissue damage, including cardiac muscle cell necrosis, liver cell degeneration, and splenic necrosis with macrophage hyperplasia. Kidney damage is also noted, with hepatic accumulation seen in some studies [52]. The most common and, thus, clinically significant toxicity is hepatotoxicity, leading to liver necrosis, apoptosis, ferroptosis, and macrophage infiltration [5].

### 4.1. Liver Toxicity

The liver cell toxicity is mediated, at least in large part, by Nrf2 inhibition due to inhibition of transcription [5,53,54]. Nrf2 is a key transcription factor involved in redox homeostasis. This important transcription factor creates a reduced cellular environment by binding to antioxidant response elements (ARE) and recruiting RNAPII to transcribe downstream antioxidant genes. Inhibition of Nrf2 has disastrous consequences for the liver, where reducing potential is an integral part of tissue function [5,53]. Inhibition of Nrf2 leads to a loss of redox balance as Nrf2 target genes handle response to oxidative stress. Indeed, activation of Nrf2 has been shown to protect against triptolide-induced hepatoxicity [53].

In one study, the authors found that triptolide exposure leads to significant reactive oxygen species (ROS) generation and oxidative stress, impairing mitochondrial function and inducing apoptosis in hepatic cells [55]. Triptolide also promotes autophagy by increasing the expression of Beclin1 and LC3II and activating autophagic flux. The study suggests that oxidative stress and autophagy are crucial in triptolide-induced hepatotoxicity, and targeting these pathways could mitigate liver damage [55].

Yet another study elucidated the role of the mitochondrial pathway in triptolide-induced cytotoxicity in human liver cells. Triptolide treatment inhibited the proliferation and induced apoptosis of L-02 cells in a concentration-dependent manner. The findings indicate that triptolide-induced apoptosis is associated with increased levels of p53 and Bax, decreased Bcl-2, loss of mitochondrial membrane potential, and cytochrome c release into the cytosol. The activation of caspases 9 and 3 further confirmed the involvement of the mitochondrial apoptotic pathway. These results provide insights into the mechanisms of triptolide-induced liver damage and suggest potential targets for therapeutic intervention [56].

The hepatotoxic effects of triptolide are attributed to multiple pathways, including CYP450-mediated metabolism, oxidative stress, excessive autophagy, apoptosis, metabolic disorders, and immune responses. Triptolide induces oxidative stress by generating ROS and impairing mitochondrial function. Additionally, autophagy and apoptosis are critical processes in triptolide-induced liver injury, involving autophagy-related proteins Beclin1 and LC3II and the pro-apoptotic proteins Bax and p53 [57].

### 4.2. Cardiotoxicity

Triptolide’s cardiotoxicity is attributed to its inhibition of the Nrf2 antioxidant system in AC16 cells. These cells are derived from primary adult ventricular tissue and can be induced to differentiate into mature cardiomyocytes. Liu and colleagues showed that triptolide caused Nrf2 inhibition [54]. Zhou and colleagues identified the Nrf2 involvement in cardiomyocyte apoptosis via the mitochondrial pathway [58]. Both ferroptosis due to lipid peroxidation and apoptosis contribute to cardiac cell death.

One study used H9C2 cells and a mitochondria-directed ROS scavenging compound to activate the Nrf2 signaling pathway and show the protective effect of alleviating ROS in triptolide-exposed cardiomyocytes [59]. Triptolide is known to cause increased p53, and it was found that this cancer-killing ability can also lead to cardiotoxicity via mitochondrial outer membrane permeabilization [60]. Another study showing that triptolide induces oxidative stress via Nrf2 inhibition used H9c2 cells to show that triptolide caused stress and mitochondrial injury. They found that transfection with an Nrf2-expressing plasmid could protect the cell from triptolide [58].

### 4.3. Nephrotoxicity

Like another cancer therapy, cisplatin, triptolide is also toxic to the kidneys, one of the most critical targets of organ toxicities that should be addressed [61,62]. It is believed that oxidative stress plays a crucial role in triptolide-induced acute nephrotoxicity. In an animal model using Sprague-Dawley rats treated with triptolide, evaluation of kidney tissues revealed structural damage as well as loss of tissues, increased apoptosis in renal cells, and decreased levels of antioxidative biomarkers, glutathione peroxidase activity, and superoxide dismutase activity, as well as an increase malonaldehyde which when elevated is a marker of oxidative stress. However, antioxidants such as vitamin C as an adjunct therapy are protective in mitigating triptolide nephrotoxicity [63].

An in vitro study using NRK-52E cells derived from rat kidney proximal tubules treated with triptolide displayed decreased antioxidant glutathione and increased ROS [62,63]. The group then transfected the cells with an Nrf2 plasmid, proving that upregulating Nrf2 could offer a protective mechanism against triptolide nephrotoxicity.

Nevertheless, another study found that triptolide induces oxidative stress in cells by increasing ROS production and depleting glutathione (GSH) levels. This oxidative stress leads to the activation of Nrf2, which then translocates to the nucleus and upregulates ARE-driven genes, such as NQO1, GCLC, and HO-1 [63]. This study again showed that overexpression of Nrf2 in NRK-52E cells significantly reduced triptolide-induced cytotoxicity, while Nrf2 knockdown worsened it, leading to increased ROS production and GSH depletion. These findings suggest that the Nrf2 pathway plays a critical protective role in counteracting triptolide-induced oxidative damage and cytotoxicity in kidney cells. This research provides insights into potential therapeutic strategies to mitigate the nephrotoxic effects of triptolide by targeting the Nrf2 pathway. The study emphasizes the importance of Nrf2 as a cellular defense mechanism and highlights the potential for developing Nrf2 activators as protective agents against triptolide-induced renal injury [63].

### 4.4. Conclusions on Toxicity

The clinical application of triptolide is significantly limited due to its broad-spectrum toxicity, particularly affecting the liver, heart, and kidneys (Figure 3). A recurring theme across multiple studies is the pivotal role of Nrf2 inhibition in mediating this toxicity. Nrf2, a crucial transcription factor for supporting redox homeostasis, is suppressed by triptolide, leading to increased oxidative stress and subsequent organ damage. This inhibition impaired the activation of antioxidant response elements, reducing cellular ability to counteract oxidative insults. The hepatotoxicity, nephrotoxicity, and cardiotoxicity induced by triptolide are all worsened by this suppression of the Nrf2 pathway.

Specifically, in the liver, the inhibition of Nrf2 leads to a loss of redox balance, contributing to oxidative stress, autophagy, and apoptosis. This is compounded by the upregulation of pro-apoptotic factors such as p53, which drive cell death. However, studies have shown that the activation of Nrf2 can effectively mitigate these toxic effects, thereby suggesting a promising therapeutic potential for Nrf2 activators in protecting against triptolide-induced hepatotoxicity.

In the heart, the triptolide-induced inhibition of Nrf2 is associated with lipid peroxidation and mitochondrial dysfunction, leading to cardiomyocyte apoptosis and ferroptosis. The cardiotoxic effects are further linked to the activation of p53, which promotes mitochondrial membrane permeabilization and cell death. However, research shows that enhancing Nrf2 activity can offer significant cardioprotection, thereby highlighting the crucial role of this pathway in supporting cardiac cell integrity under triptolide treatment.

Triptolide similarly affects the kidneys through Nrf2 inhibition, increasing ROS production and GSH depletion. This oxidative stress damages renal cells and reduces their viability. Overexpression of Nrf2 in kidney cells has been shown to counteract these effects, underscoring the protective role of Nrf2 in renal tissues.

The consistent findings from various studies underscore the pivotal role of Nrf2 inhibition in triptolide toxicity. Strategies aimed at boosting Nrf2 activity, whether through pharmacological activation or genetic upregulation, hold significant promise to mitigate the adverse effects of triptolide. Future research should prioritize developing and refining such interventions to expand triptolide’s therapeutic window and clinical utility.

## 5. Pharmacokinetics

### 5.1. Absorption

Triptolide demonstrates rapid absorption after oral and intravenous (i.v.) administration. In a study involving male Sprague-Dawley rats, the concentration of triptolide in plasma peaked within 15 min after oral administration of doses ranging from 0.6 to 2.4 mg/kg, indicating swift gastrointestinal absorption [52]. The elimination half-life of triptolide after oral administration varied from 16.81 to 21.70 min, confirming its rapid absorption and clearance from the system [52]. Similarly, intravenous administration resulted in rapid absorption, and the kinetic properties of triptolide fitted a one-compartment model, suggesting straightforward pharmacokinetics [52].

Oral bioavailability of triptolide varies, reported at 63.9% for a 1 mg/kg dose and 72.08% for a 0.6 mg/kg dose in rats. However, intravenous administration of 1 mg/kg results in complete serum clearance within 2 h, with a plasma half-life of 11.4 min, while the same oral dose is cleared in 3 h, having a plasma half-life of 25.2 min. Another study noted a plasma half-life of 21.7 min for an oral dose of 0.6 mg/kg. These pharmacokinetic challenges, combined with extensive “first pass” hepatic metabolism (in the case of oral administration) involving microsomes and various CYP450 isoforms and interaction with P-glycoprotein leading to intestinal efflux, complicate oral dosing despite good net absorption [52,64].

Triptolide readily crosses the blood–brain barrier (BBB), allowing triptolide to exert potent therapeutic effects in various central nervous system conditions, including Alzheimer’s disease, ischemic brain injuries, and gliomas. This property is crucial for its potential clinical applications to these diseases, as it enables the drug to reach therapeutic concentrations in the brain and modulate disease processes directly within the central nervous system [50,65,66,67,68]. Additionally, its small size and hydrophobic character make it highly likely that triptolide can cross the placenta. Although specific studies of teratogenicity in mammals are lacking, triptolide is teratogenic in zebrafish [69]. This makes the dosing of pregnant females with triptolide a concern.

### 5.2. Distribution

Triptolide is distributed rapidly throughout various tissues post-administration. Following oral administration at a dose of 1.2 mg/kg, triptolide was detected in high concentrations across all tested tissues, including the liver, spleen, lungs, kidneys, and heart, within 5 min post-dose [52]. The distribution closely followed the plasma concentration curve, suggesting no significant tissue accumulation. This pattern was consistent with findings from other studies where triptolide demonstrated similar rapid distribution in tissues after intravenous administration [52]. In addition, PLGA microspheres containing triptolide injected intra-articularly in a rat model of rheumatoid arthritis also showed effective distribution, with notable therapeutic effects on the targeted tissues [70].

### 5.3. Metabolism

Triptolide undergoes extensive metabolism, as indicated by the low recovery of the parent compound in excretory pathways. In rats, less than 1% of the administered dose was recovered as unchanged triptolide in bile, urine, or feces within 48 h of administration, signifying significant metabolic transformation [52]. The primary sites of metabolism were identified as the liver, where both cytochrome P450 enzymes and P-glycoprotein played crucial roles in the metabolic pathways of triptolide, potentially contributing to its hepatotoxicity [64]. Moreover, studies utilizing LC-MS/MS methods confirmed the presence of multiple metabolites in biological samples, underscoring the complexity of triptolide’s metabolic processes [71].

### 5.4. Excretion

Triptolide and its metabolites are excreted primarily through biliary and urinary pathways, although in minimal amounts as the unchanged drug. Detailed studies on the excretion of tritiated triptolide in rats revealed that only 1.92% of the administered dose was recovered in feces, 3.98% in bile, and 3.81% in urine within 24 h [72]. This extensive metabolism and subsequent excretion as metabolites rather than the parent compound further highlight the rapid metabolic processing triptolide undergoes post-administration.

### 5.5. Pharmacokinetic Studies in Human Subjects

The first human phase I study of Minnelide (a water-soluble prodrug of triptolide) in patients with advanced gastrointestinal cancers provided additional insights into the drug’s pharmacokinetics [73]. “Minnelide was rapidly converted to triptolide in all patients, with peak triptolide levels observed shortly after... the 30-min infusion (T_max_ range: 0.54 to 0.65 h). The half-life of triptolide was approximately 1 h (median: 0.76 h; range: 0.36 to 2.3 h), with complete clearance in most patients by 3 h.” [73]. This study indicated similar pharmacokinetic properties to those observed in animal models, including rapid absorption, widespread tissue distribution, extensive metabolism, and excretion predominantly as metabolites [73]. This consistency across species underlines the predictable pharmacokinetic behavior of triptolide and its derivatives in clinical settings.

### 5.6. Conclusions on Pharmacokinetics

In conclusion, triptolide exhibits rapid absorption, extensive distribution, significant metabolism, and minimal excretion as an unchanged drug in both animal models and human studies. These pharmacokinetic properties are crucial for understanding its therapeutic potential and associated toxicities: the compound’s rapid and extensive hepatic metabolism and poor water solubility result in suboptimal pharmacokinetic characteristics. Further studies are warranted to elucidate the precise metabolic pathways and the role of specific enzymes in triptolide metabolism, which will aid in optimizing its clinical use and mitigating adverse effects. Additionally, nanotechnology-based delivery systems and targeted formulations are being investigated to enhance the pharmacokinetic properties of triptolide and reduce its toxicity [74,75,76,77].

## 6. Strategies to Improve Triptolide’s Pharmacological Profile

Researchers have explored various strategies to address the limitations of triptolide’s pharmacokinetic and toxicity profiles, including developing derivatives and prodrugs. One such approach involves the creation of water-soluble prodrugs that can be metabolized in vivo to release active triptolide, thereby enhancing bioavailability and reducing systemic toxicity [78]. For instance, Minnelide (Figure 2), a water-soluble prodrug of triptolide, has demonstrated promising outcomes in preclinical studies by achieving higher therapeutic concentrations while minimizing adverse effects [79]. This prodrug strategy capitalizes on converting Minnelide to active triptolide by esterase enzymes in the body, facilitating improved drug delivery and efficacy [79].

### 6.1. Structural Modification

#### 6.1.1. Glutriptolides

Another method to improve triptolide’s pharmacological profile is to generate glucose–triptolide conjugates. The glucose–triptolide conjugates, known as glutriptolides, exploit the overexpression of glucose transporters (GLUTs) in cancer cells. These transporters, particularly GLUT1 and GLUT3, are upregulated in tumor cells to meet their increased glucose demand, a phenomenon associated with the Warburg effect. By conjugating triptolide with glucose, researchers aim to selectively target cancer cells while sparing normal cells, thereby reducing systemic toxicity [3].

Glutriptolide-2, a second-generation glucose–triptolide conjugate, has shown promising results in preclinical studies. It has improved stability in human serum, greater selectivity for cancer cells, and increased potency against hypoxic cancer cells compared to normoxic conditions. This enhanced efficacy under hypoxia is particularly relevant, as the hypoxic microenvironment within tumors often contributes to chemoresistance. Glutriptolide-2 showed sustained antitumor activity in vivo, significantly prolonging survival in a metastatic prostate cancer model [80]. Additionally, glutriptolide-2 shows higher water solubility than triptolide, easing administration and enhancing bioavailability, making it more practical for clinical development. Selective uptake by cancer cells through GLUTs ensures a higher drug concentration within the tumor, maximizing therapeutic efficacy while minimizing systemic toxicity [3,80].

The synthesis of glutriptolides involves linking triptolide to glucose at the C14 hydroxyl group, the most amenable site on the triptolide skeleton. Different linkages, including succinate, acetal, and ether bonds, have been explored to improve the release of active triptolide within the tumor cells. Glutriptolides with succinate linkers, such as glutriptolide-2, show enhanced antiproliferative activity due to efficient cleavage by cellular esterases, leading to the release of triptolide inside the cancer cells [3].

In vitro studies have shown that glutriptolide-2 induces the degradation of the catalytic subunit of RNAPII through a two-step mechanism. Initially, glutriptolide-2 is converted to triptolide which binds XPB, inhibiting its DNA-dependent ATPase activity, which prevents the initiation of transcription by RNAPII. Subsequently, this binding triggers the proteasome-mediated degradation of RNAPII, amplifying the transcriptional inhibition and leading to cancer cell death [80]. We will go into more detail on the mechanism in a later section.

In vivo, glutriptolide-2 has shown remarkable tumor control. In a prostate cancer metastasis model, treatment with glutriptolide-2 resulted in significantly lower tumor burdens and prolonged survival compared to untreated controls and those treated with unmodified triptolide. This sustained antitumor effect is attributed to the prolonged release of active triptolide within the tumor cells, which maintains therapeutic concentrations over an extended period [3].

Moreover, glutriptolide-2 shows a higher water solubility than triptolide, overcoming one of the significant limitations of triptolide as a drug candidate. The increased solubility eases its administration and enhances its bioavailability, making it a more workable possibility for clinical development [80].

#### 6.1.2. Other C-14-βOH Conjugates

Beyond glutriptolides, various other C-14 β hydroxyl group modifications have been explored and developed. Noteworthy examples include PG490-88 (aka F60008), a 14C-succinate salt derivative of triptolide designed to enhance water solubility and oral bioavailability, WilGraf, a 14C-tert-Butyl carbonate triptolide derivative created by Pharmagenesis, and Minnelide, a 14C-O-phosphonooxymethyl derivative salt developed by researchers at the University of Minnesota [1].

These compounds have met with varying levels of success. For example, research indicates that Minnelide effectively eliminates CD133+ cancer stem cells in pancreatic cancer, which are often implicated in tumor recurrence and metastasis [81]. Additionally, Minnelide has been shown to inhibit NER and act synergistically with oxaliplatin to overcome resistance in cancer [20]. In its first Phase I clinical trial, Minnelide was shown to be effective against pancreatic and gastric cancer. The results of that study were published very recently, in 2024 [73].

Wilgraf, in contrast, has dropped out of development, and it is difficult to find mention of this triptolide conjugate anywhere [1]. In preclinical studies, PG490-88 demonstrated promising results, including the ability to inhibit alloreactive T cell expansion, which is crucial for preventing graft-versus-host disease (GVHD) in preclinical studies [82]. However, the development of PG490-88 has proceeded in fits and starts over the years. It originally began Phase I clinical trials in 2003 in Europe. Unfortunately, these trials were not completed [83]. In 2009, the results of a Phase I study of PG490-88 were published. The disappointing conclusions of the study were that “Pharmacokinetic studies showed high inter-individual variability and rendered F60008 a far from optimal derivate of triptolide [84]”.

Other derivations at this position have been explored, including (14S)-14,21-epoxytriptolide, epitriptolide, and even PEGylated triptolide prodrugs for self-assembling nanoparticles [83,85]. Again, although varying levels of success have been observed, none of these agents has successfully advanced in clinical trials.

#### 6.1.3. Additional Derivations of Triptolide

LLDT-8 (aka (5R)-5-hydroxytriptolide) is a notable derivative of triptolide that has enhanced therapeutic efficacy and reduced toxicity developed for the treatment of inflammatory and autoimmune disease, especially rheumatoid arthritis. LLDT-8 was developed in China by researchers at the Shanghai Institute of Materia Medica and has progressed through Phase I and Phase II clinical trials [86].

One of the important features of LLDT-8 is its potent immunosuppressive activity. Studies have demonstrated that LLDT-8 primarily targets some subsets of activated T cells, inhibiting their proliferation and reducing the production of pro-inflammatory cytokines such as interferon-γ (IFN-γ) [87]. LLDT-8 has also shown efficacy beyond autoimmune and inflammatory diseases by preventing GVHD in murine models by expanding regulatory T cells, which are essential for maintaining immune tolerance [88].

A 2023 publication reported the findings of a Phase II clinical trial investigating LLDT-8 in individuals infected with HIV [40]. The study demonstrated that LLDT-8 improved CD4 recovery and reduced inflammation in long-term suppressed immunological non-responders. These individuals had previously experienced inadequate CD4 T cell recovery despite sustained virological suppression, and LLDT-8 offered a potential solution to this issue [37]. Another clinical trial of LLDT-8 for rheumatoid arthritis was completed in 2017 in China, but the results of this study have not been published [8].

LLDT-8 represents a significant advancement in the therapeutic application of triptolide derivatives, particularly for treating inflammatory and autoimmune diseases such as rheumatoid arthritis. However, while LLDT-8 has progressed through clinical trials and has shown promising results, further studies are necessary to evaluate its therapeutic potential fully and to confirm its safety and efficacy. The unpublished results from the rheumatoid arthritis trial and continued research into its mechanisms of action and long-term effects will be essential in determining its future role in clinical practice. Overall, LLDT-8 exemplifies the potential of modified triptolide derivatives to balance efficacy with reduced toxicity, providing a foundation for novel treatment strategies in immunological and inflammatory diseases [6,37].

### 6.2. Delivery Systems

Many studies [74,75,76,78,89] of nanotechnology-based carrier systems have highlighted the various innovations in triptolide delivery. These systems (Table 1), including nanoparticles, micelles, liposomes, and exosomes, significantly improve drug solubility, stability, and targeting [78]. These advanced delivery systems enhance triptolide’s overall therapeutic efficacy, making it a more workable option for treating various diseases.

Liposome hydrogel patches are a significant advancement in the localized delivery of triptolide. A recent study investigated the pharmacokinetic and pharmacodynamic effects of a triptolide-loaded liposome hydrogel patch administered via microneedles in rats with collagen-induced arthritis. The results showed that this delivery system enhanced the anti-inflammatory effects and improved the bioavailability of triptolide, making it a promising approach for localized treatment of inflammatory conditions [90]. This innovation mitigates systemic toxicity and provides sustained and controlled drug release, highlighting its potential in clinical applications.

Polymeric micelles and nanoparticles have also shown promise in enhancing triptolide’s therapeutic efficacy. Polymeric micelles improve the solubility and stability of triptolide, leading to increased therapeutic efficacy, particularly in cancer treatment. Additionally, multiple-coated PLGA nanoparticles have been developed to load triptolide, demonstrating significant attenuation of injury in a mouse model [91,92]. These delivery systems provide targeted and sustained drug release, reducing systemic toxicity and enhancing therapeutic outcomes in various disease models.

Stimuli-responsive nanoparticles are an innovative approach to the targeted delivery of triptolide. These nanoparticles can release their payload in response to specific environmental triggers, such as pH or redox conditions. For example, a novel CD44-targeting and pH/redox-dual-stimuli-responsive core-shell nanoparticle system has been developed to combat breast cancer growth and lung metastasis [89]. This innovative delivery system allows for controlled drug release, enhancing the selectivity and efficacy of triptolide in cancer therapy. The ability to respond to the tumor microenvironment ensures that the drug is released specifically at the tumor site, minimizing off-target effects and improving patient outcomes.

Exosome-based delivery systems offer a biocompatible and efficient platform for the delivery of triptolide. Exosomes are small vesicles that can encapsulate and protect drugs, helping their transport across biological barriers. In vitro studies have demonstrated that triptolide-loaded exosomes can significantly inhibit the proliferation and induce apoptosis of human cancer cells [75]. This approach uses the natural targeting abilities of exosomes to deliver triptolide directly to cancer cells, enhancing its therapeutic efficacy while reducing systemic toxicity. Thus, exosome-based delivery systems are a promising strategy for treating various cancers.

Microemulsions and hydrogel formulations have also been explored to improve the delivery of triptolide. Triptolide-loaded hydrogel-thickened microemulsions have been evaluated for their safety and efficacy in vivo, offering a unique approach to improving the stability and bioavailability of triptolide [93]. These formulations provide a controlled release mechanism, ensuring sustained therapeutic levels of triptolide in the target tissues. Thus, hydrogels and microemulsions are versatile and practical strategies for delivering triptolide in various clinical settings.

Targeted nano-drugs have been developed to enhance the delivery of triptolide to specific tissues. For instance, HER2-targeted nano-drugs loaded with triptolide have shown superior efficacy in delivering the drug directly to tumor cells [74]. This targeted approach reduces systemic toxicity and enhances therapeutic outcomes by ensuring that triptolide is concentrated at the tumor site. Similarly, kidney-targeted delivery systems for triptolide encapsulated in nanoparticles have been explored to treat renal diseases [76]. These systems precisely target the kidneys, minimizing off-target effects and improving the therapeutic index of triptolide.

Solid dispersions and liquid crystals have been investigated as innovative delivery systems for triptolide. The mechanochemical preparation of triptolide-loaded self-micelle solid dispersions has enhanced oral bioavailability and improved antitumor activity in cancer treatment [77]. Additionally, cubic and hexagonal liquid crystals have been studied as drug carriers for the transdermal delivery of triptolide [94]. These novel formulations bypass the gastrointestinal tract and first-pass metabolism, offering an effective route of administration for triptolide. Transdermal formulations are advantageous for treating rheumatoid arthritis.

For hepatocellular carcinoma and rheumatoid arthritis, targeted delivery systems have been developed to enhance the therapeutic efficacy of triptolide. Galactosylated chitosan nanoparticles have been designed to overcome hepatocellular carcinoma by delivering triptolide specifically to liver cells [95]. Similarly, a novel carboxylated chitosan-based triptolide conjugate has shown promise in treating rheumatoid arthritis, providing targeted and sustained drug release to inflamed joints [96]. These targeted approaches ensure that triptolide is delivered precisely to the diseased tissues, enhancing its therapeutic effects while minimizing systemic toxicity.

Lipid emulsions have also been explored for the delivery of triptolide. Triptolide-loaded lipid emulsions have been studied for their pharmacokinetics and tissue distribution in mice, showing significant accumulation in the pancreas [97]. This formulation enhances triptolide’s delivery and therapeutic potential for pancreatic diseases, offering a sustained release mechanism that keeps therapeutic drug levels in the target tissues.

### 6.3. Microbiome

Emerging research has demonstrated that the gut microbiota plays a significant role in the metabolism of various drugs, including triptolide, an anti-inflammatory and anticancer compound known for its potent toxicity. Recent studies have shown that gut bacteria can metabolize triptolide into distinct metabolites, which may contribute to both its therapeutic effects and toxic side effects.

In one study, Peng et al. (2020) demonstrated that the gut microbiota metabolizes triptolide into four major metabolites, including two novel dehydrogenated metabolites that had not been previously reported in liver metabolism [98]. These findings suggest that gut bacteria have unique metabolic pathways that can significantly alter the bioavailability and toxicity of triptolide. Furthermore, the inactivation of the gut microbiota led to a marked reduction in triptolide metabolism, indicating the critical role of intestinal bacteria in this process [98]. This highlights the potential for targeting gut microbiota to mitigate the toxic side effects of triptolide, such as hepatotoxicity. Similarly, Zhao et al. (2021) have explored the link between gut microbiota and testicular dysfunction in a mouse model exposed to triptolide [99]. Their research revealed that polyamine metabolism, influenced by gut microbiota, plays a protective role in testicular health, with specific bacteria like *Parabacteroides distasonis* showing potential in reducing triptolide-induced testicular injury [99]. This research underscores the broader impact of gut microbiota on triptolide toxicity and suggests that manipulating microbial populations could provide a novel approach to improving the drug’s safety profile.

In another vital study, Huang et al. (2020) demonstrated that gut microbiota-derived propionate, a short-chain fatty acid, significantly reduced triptolide-induced hepatotoxicity [100]. Mice with depleted gut microbiota exhibited exacerbated liver injury, whereas supplementation with propionate restored metabolic homeostasis, decreased inflammatory markers, and reduced long-chain fatty acid accumulation. These findings emphasize the protective role of gut microbiota in maintaining liver health and further highlight how modulating specific gut-derived metabolites like propionate can mitigate triptolide toxicity [100]. The study illustrates the critical role of gut microbiota-derived metabolites like propionate in protecting against triptolide-induced hepatotoxicity, suggesting that enhancing the production of such beneficial metabolites could serve as a promising strategy to reduce the drug’s toxic effects. Targeting specific microbial pathways may offer a novel therapeutic approach to improving triptolide’s safety and efficacy.

Given the growing understanding of gut microbiota’s involvement in drug metabolism, these findings provide new avenues for mitigating triptolide toxicity and enhancing its therapeutic efficacy. Future studies should explore how modulation of gut bacteria could lead to safer, more effective treatments involving triptolide. Understanding the specific microbial species and their metabolic contributions could also allow for the development of personalized microbiota-based therapies tailored to individual patient needs. This approach may broaden triptolide’s clinical utility while minimizing its toxic side effects.

## 7. Protein Targets of Triptolide

Triptolide and derivatives impact many pathways in inflammation, immunosuppression, cell death, and proliferation through direct or indirect interactions with numerous proteins involved. Here, we highlight selected proteins with experimental evidence for direct interactions (Table 2).

### 7.1. Polycystin-2 (PC-2)

PC-2 is a calcium channel highly expressed in the ovary, kidney, testes, and small intestine. In the kidney, it is expressed in multiple parts of the nephron. The channel facilitates calcium release from the ER [47]. PC-2 may be a helpful triptolide target to treat autosomal dominant polycystic kidney disease. Leuenroth et al. (2008) showed that triptolide can significantly decrease cyst formation in a Pkd1-deficient mouse model [45]. Their study revealed that triptolide’s effectiveness in mitigating cystic burden is likely mediated through its ability to restore PC-2-mediated calcium release, inhibiting the early proliferative phases of cyst growth [45]. In their experiments, triptolide treatment led to a marked reduction in the number and size of renal cysts and improved renal function. The study used a kidney-specific Cre-loxP system to induce Pkd1 deletion, which mimicked the rapid cystogenesis observed in neonatal mice. The results indicated that triptolide’s antiproliferative effects on cyst epithelium were primarily seen during the first stages of cyst formation. However, the treatment did not significantly affect the expansion of already-established cysts [45]. This is an exciting route for triptolide development.

### 7.2. dCTP Pyro-Phosphatase 1 (DCTPP1)

DCTPP1, involved in nucleotide metabolism, has a low affinity for triptolide, with K_i_ of 168 µM and K_d_ of 44 ± 4 µM. The binding of triptolide to DCTTP1 is reversible [110]. Triptolide and its analogs block the pyrophosphatase action by DCTTP1 on the active metabolite of decitabine. Decitabine is a commonly administered chemotherapy drug used to treat hematologic malignancies, and it is an antimetabolite. 5-aza-dCTP, the active metabolite of decitabine, is generated via reaction with DCTPP1, thus enabling increased drug integration into the genome. This increases DNA methyltransferase degradation, a boost in demethylation, and alterations in gene expression programs [111]. The level of DCTPP1 expression correlated with the sensitivity to DNA methyltransferase inhibitors. These findings suggest a role for DCTPP1 in the resistance of cancer cells to nucleoside analog DNA methyltransferase inhibitors. Therefore, combining triptolide or its analogs with existing nucleoside analog DNA methyltransferase inhibitors holds promise for further exploration in preclinical and clinical trials for human cancers [7,101,110].

### 7.3. Peroxiredoxin I (Prx I)

Prx I is a multifunctional protein that serves dual roles as an antioxidant and a chaperone, playing a critical role in cancer development and inflammation. Its antioxidant activity primarily involves the catalysis of hydrogen peroxide (H_2_O_2_) removal, while its chaperone function prevents protein aggregation under stress conditions. Triptolide, a potent anticancer compound, has been found to selectively inhibit the chaperone activity of Prx I without affecting its antioxidant activity. This selective inhibition is achieved through the covalent binding of triptolide to cysteine residues, specifically Cys83 and Cys173, which are crucial for maintaining Prx I’s oligomeric structure necessary for its chaperone function [113].

The interaction between triptolide and Prx I triggers the dissociation of Prx I oligomers, leading to a dose-dependent impairment of its chaperone activity. Despite this inhibition, the potent antiproliferative effects of triptolide on cancer cells are not solely explained by the inhibition of Prx I’s chaperone function, suggesting that other molecular targets and pathways are involved [113]. The covalent binding mechanism of triptolide to cysteine residues in Prx I is like its binding mechanism to XPB, underscoring its specific and potent interaction with Prx I [113]. This interaction is crucial for the functional switching of Prx I from a peroxidase to a molecular chaperone under oxidative stress conditions [115].

Moreover, peroxiredoxins, including Prx I, have been identified as crucial chaperone reservoirs in various organisms, such as *Leishmania infantum*, where they act to prevent protein aggregation [116]. As a family of peroxidases, peroxiredoxins are essential in protecting cells from oxidative stress and maintaining cellular homeostasis [117]. They are known to undergo functional changes, such as transitioning from peroxidase to molecular chaperone activity upon overoxidation of their catalytic cysteine residues [118]. Additionally, peroxiredoxins have been implicated in diverse cellular functions, including cell cycling, gene expression, and apoptosis, and are suggested to be involved in diseases such as cancer [119].

In conclusion, Prx I shows dual functionality as an antioxidant enzyme and a molecular chaperone, with triptolide selectively inhibiting its chaperone activity through covalent binding to specific cysteine residues. This inhibition impairs Prx I’s chaperone function in a dose-dependent manner but does not solely account for triptolide’s antiproliferative effects on cancer cells. The broader roles of peroxiredoxins in cellular processes and diseases further emphasize their importance in keeping cellular homeostasis and their potential as therapeutic targets.

### 7.4. A-Disintegrin and Metalloprotease-10 (ADAM10)

ADAM10 is a metalloproteinase crucial for membrane protein cleavage, significantly influencing cellular signaling, cell growth, and cell migration. The overexpression of ADAM10 in various cancers and its role in promoting tumor growth make it an attractive target for cancer therapy [120,121]. Triptolide can reduce the expression of ADAM10 in various cancer cells [109]. In addition, the direct interaction of triptolide and ADAM10 was demonstrated through affinity chromatography and mass spectrometry [109]. Interestingly, further investigations revealed that siRNA-mediated knockdown of ADAM10 in MCF-7 cells, followed by triptolide treatment, significantly reduced cell growth [109].

### 7.5. TAK1 Binding Protein (TAB1)

Transforming growth factor β-activated kinase 1 (TAK1) is a crucial mediator in several intracellular signaling pathways, including those involved in inflammatory and immune responses. TAK1’s activity is regulated by interacting with TAK1-binding proteins such as TAB1, TAB2, TAB3, and TAB4 [122]. Among these, TAB1 plays a significant role by directly activating TAK1 and promoting its kinase activity. Triptolide targets TAB1 to inhibit the TAK1-TAB1 complex formation, thereby suppressing the downstream signaling pathways [7,101,112].

In 2014, Shen and colleagues identified TAB1 as a binding target of triptolide using a monoclonal antibody approach [7,101,112]. This triptolide binding disrupts the formation of the TAK1-TAB1 complex, resulting in reduced kinase activity of TAK1. The dissociated TAK1 cannot phosphorylate its downstream targets, MKK3/6 and MKK4, which are critical for activating p38 and JNK1/2. This inhibition subsequently reduces the expression and release of several inflammatory cytokines, including TNF-α, IL-1β, and IL-6, which are critical players in inflammatory and immune responses [101,112].

Detailed studies into the binding mechanism of triptolide to TAB1 revealed that specific amino acid sequences within TAB1 are crucial for this interaction. Deletion mutants of TAB1, particularly those lacking amino acids 373 to 418, showed significantly reduced binding affinity for triptolide. This region is also necessary for the formation of the p38α-TAB1 complex, suggesting that triptolide binding induces structural changes in TAB1 that prevent its interaction with TAK1 [112].

Moreover, the inhibitory effect of triptolide on TAB1-mediated TAK1 activation has implications beyond inflammation. While suppressing NF-κB and AP1 pathways by inhibiting the TAK1-TAB1 interaction explains the anti-inflammatory effects of triptolide, it does not fully account for its antitumor activity. This suggests that triptolide may have other targets or mechanisms contributing to its anticancer properties [7,112].

### 7.6. The DNA–PK Complex Kinase Subunit (DNA–PKcs)

DNA-PKcs is crucial for DNA damage repair (DDR), particularly in repairing DNA double-strand breaks (DSBs) through non-homologous end joining (NHEJ). It forms a holoenzyme with the Ku70/Ku80 heterodimer, which helps recognize and bind to DSBs. Additionally, DNA-PKcs is involved in several cellular processes beyond DSB repair, such as replication stress response, cell cycle checkpoints, telomere maintenance, senescence, and autophagy. These diverse roles highlight its importance in cellular homeostasis and response to genomic insults [102].

Due to its pivotal role in DDR, DNA-PKcs is a target for cancer therapy. Inhibitors of DNA-PKcs can enhance the efficacy of radiotherapy by sensitizing cancer cells to DNA damage. This approach holds promise for developing more effective cancer treatments. Triptolide’s interaction with DNA-PKcs significantly affects the DNA damage response. Upon binding to DNA-PKcs, triptolide inhibits the autophosphorylation of DNA-PKcs at the Ser2056 residue. This inhibition prevents DNA-PKcs from recruiting essential DNA repair factors, such as p53-binding protein 1 (53BP1), to the site of DNA breaks. Consequently, the DNA repair process is disrupted, accumulating DNA damage within the cell [7].

The inhibition of DNA-PKcs autophosphorylation by triptolide also affects the regulation of cell cycle checkpoints. DNA-PKcs is known to play a role in the G2/M checkpoint, and its inhibition can lead to prolonged cell cycle arrest, contributing to cell death through apoptosis. This mechanism is particularly relevant in cancer cells, where the accumulation of DNA damage and failure to repair can lead to enhanced sensitivity to other treatments, such as chemotherapy and radiotherapy [7,102].

### 7.7. Xeroderma Pigmentosum B (XPB or ERCC3 Gene Product)

The primary molecular target of triptolide is the XPB protein, a vital subunit of the TFIIH complex involved in both transcription initiation by RNAPII and NER [17]. Through its interaction with XPB, triptolide exerts multifaceted inhibitory effects on both transcription and DNA repair processes, underscoring its therapeutic potential in cancer treatment [15,16,21].

XPB plays a critical role in the transcription initiation by RNAPII, functioning as a DNA helicase that unwinds DNA at gene promoters, facilitating the formation of the transcription bubble [123]. Triptolide binds to XPB at cysteine 342, a conserved residue in eukaryotes, covalently attached through its 12,13-epoxide functional group [16,21]. This cysteine residue is next to the Walker A motif, a conserved phosphate-binding site crucial for ATPase activity in helicases. By occluding this site, triptolide abolishes the ATPase activity of XPB [21]. Consequently, the melting of gene promoters necessary for transcription initiation is impeded, leading to RNAPII stalling at promoters [15,16].

The inhibition of transcription by triptolide involves a biphasic mechanism. Initially, RNAPII transcription is hindered by the loss of XPB activity, causing stalling at promoters [15]. During this phase, triptolide-induced aberrant activation of XPB alters the phosphorylation patterns of the Rbp1 subunit of RNAPII, specifically inhibiting the phosphorylation of serine 2 and serine 7, which are crucial for promoter escape and transcription elongation. As the process continues, activated CDK7 hyper-phosphorylates the stalled RNAPII subunit Rpb1 at serine 5, which subsequently leads to the engagement of p44’s E3 ubiquitin ligase activity on lysine 7 in the C-terminal domain. This results in the proteasome-mediated degradation of RNAPII, causing sustained transcription inhibition [15,19]. While the clinical relevance of the second phase of inhibition remains uncertain, it has been observed at higher doses and longer time scales [15].

In addition to its impact on transcription, triptolide significantly disrupts the NER pathway, which is crucial for repairing bulky DNA lesions induced by ultraviolet (UV) light and chemical agents. XPB’s helicase activity is essential for the initial melting of the DNA repair bubble, which is subsequently expanded by another TFIIH subunit, XPD [124]. Triptolide’s inhibition of XPB’s ATPase activity prevents this initial melting, thereby abolishing the NER activity of TFIIH [16]. This action becomes particularly significant when triptolide is co-administered with DNA-damaging agents such as platinum compounds, as the inhibition of NER hampers the repair of platinum-induced DNA lesions, leading to apoptosis [11,12,20,32].

## 8. Important Downstream Effectors of Triptolide

### 8.1. p53

One of the primary mechanisms by which triptolide exerts its anticancer effects is by enhancing p53 activity. For example, in human laryngocarcinoma cells, triptolide stabilizes p53 by preventing its degradation, leading to growth inhibition and apoptosis. This suggests that triptolide’s ability to stabilize p53 allows it to perform its tumor-suppressive roles more effectively [125]. RNAPII and TFIIH are required for the transcription of p53 target genes [126]. Although triptolide broadly inhibits transcription, levels of p53 increase in cells after triptolide administration, highlighting the counterintuitive and complex effects of triptolide.

Triptolide also affects the MDM2-p53 interaction, which is critical in cancers where MDM2 is overexpressed. By inhibiting MDM2, a negative regulator of p53, triptolide enhances p53’s apoptotic signaling pathways, promoting cell death in tumor cells. This mechanism is essential in cancers where restoring p53 function is crucial to treatment outcomes [34,127].

Furthermore, triptolide’s ability to inhibit MDM2 and induce apoptosis has been studied in acute leukemia models. In these settings, triptolide not only inhibits MDM2 but also enhances the intrinsic apoptotic pathway by modulating critical proteins involved in mitochondrial-mediated apoptosis. This dual mechanism is crucial in cancers like AML, where restoring p53 function is an important therapeutic goal [128].

Additionally, triptolide-induced activation of the p38 MAPK pathway boosted p53’s transcriptional activity by promoting phosphorylation, thereby enhancing tumor-suppressive functions. The activation of p38 MAPK by triptolide was also linked to an increase in p53-mediated apoptotic signaling, contributing to the overall antitumor effects observed in several cancer models. Through this signaling pathway, triptolide further strengthens p53’s ability to suppress tumor growth [34,129].

Triptolide’s ability to target both p53-dependent and independent pathways positions it as a potent anticancer agent, warranting further research for its optimal use in specific cancers. By stabilizing p53, inhibiting the MDM2-p53 interaction, and enhancing mitochondrial-mediated apoptosis, triptolide effectively restores and amplifies p53’s tumor-suppressive functions in various cancer models. Furthermore, triptolide’s activation of the p38-MAPK pathway underscores its multiple impacts on p53, further boosting p53’s activity and driving apoptotic processes. These combined effects highlight triptolide’s potential in overcoming cancer cell resistance, particularly in malignancies characterized by dysregulated p53 pathways. As understanding of its molecular targets continues to grow, triptolide may offer promising therapeutic avenues, particularly in cancers where restoring p53 function is essential to achieving successful treatment outcomes [127].

### 8.2. NF-κB

NF-κB is a critical transcription factor that regulates immune responses, inflammation, and cell survival, and its activation typically occurs through a well-characterized signaling cascade. This cascade involves the phosphorylation and degradation of IκB proteins, which sequester NF-κB in the cytoplasm. Once IκB is degraded, NF-κB translocates to the nucleus, binding to specific DNA sequences to initiate transcription of various genes, including those involved in inflammation and apoptosis [130,131]. NF-κB is inhibited by triptolide at the transcription level and is believed to be a consequence of triptolide’s interaction with XPB and subsequent inhibition of TFIIH [29,131].

Triptolide disrupts the phosphorylation of IκB proteins, essential for NF-κB activation. In addition to blocking the phosphorylation of IκB, triptolide has been reported to interfere with the activity of the IκB kinase (IKK) complex, which is responsible for initiating the degradation of IκB and allowing the expression of NF-κB target genes. Studies have demonstrated that, as a consequence, triptolide can downregulate the expression of antiapoptotic genes regulated by NF-κB, such as Bcl-2 and Bcl-xL, which further supports its role in inhibiting NF-κB-mediated transcriptional activation [25,132,133]. RNAPII and TFIIH are required for the transcription of NF-κB target genes [134,135]. Therefore, inhibition of NF-κB likely results from triptolide binding to XPB.

### 8.3. Nrf2

One of the primary mechanisms by which triptolide induces toxicity through oxidative stress is characterized by the excessive generation of ROS. The transcription factor Nrf2 plays a pivotal role in defending cells against oxidative stress by regulating the expression of antioxidant and detoxifying enzymes. Mounting evidence suggests that activation of the Nrf2 pathway can protect against triptolide-induced toxicities, making it a critical therapeutic target.

Triptolide-induced hepatotoxicity is a significant limitation to its therapeutic use. Studies show that oxidative stress and mitochondrial dysfunction are substantial contributors to triptolide-induced liver injury. Triptolide causes depletion of intracellular antioxidants, such as GSH, leading to lipid peroxidation and apoptosis of hepatocytes [53]. Nrf2 activation has been shown to protect against this hepatotoxicity. For example, overexpression of Nrf2 in HepG2 cells reduced oxidative stress. Additionally, it promoted the expression of detoxifying enzymes like NAD(P)H quinone oxidoreductase 1 (NQO1), heme oxygenase-1 (HO-1), and glutamate cysteine ligase catalytic subunit (GCLC), which are critical in maintaining redox balance [53]. Pharmacological activation of Nrf2 using sulforaphane (SFN) mitigated triptolide-induced liver damage in mice by enhancing the expression of these antioxidant enzymes [53]. Conversely, Nrf2 knockdown exacerbated the hepatotoxic effects of triptolide, highlighting Nrf2’s protective role in liver tissues [53].

The cardiotoxic effects of triptolide are primarily driven by oxidative stress and mitochondrial dysfunction. In rat cardiomyocyte H9c2 cells, triptolide has been shown to induce mitochondrial fragmentation and excessive ROS production, leading to cardiomyocyte apoptosis. Activation of the Nrf2 pathway offers a protective mechanism against these effects. A study involving MitoQ, a mitochondria-targeted antioxidant, demonstrated that triptolide-induced cardiotoxicity could be significantly alleviated by activating the p62-Nrf2 pathway. MitoQ treatment led to the upregulation of Nrf2 and its downstream antioxidant genes, such as HO-1 and NQO1, which helped to restore mitochondrial function and improve cell survival [59]. The knockdown of Nrf2 in this model eliminated the protective effects of MitoQ, confirming that Nrf2 is crucial in mitigating the cardiotoxic effects of triptolide [59].

Triptolide also causes nephrotoxicity, primarily mediated by oxidative stress. The Nrf2 pathway plays a protective role in the kidney, where it helps to counteract oxidative damage induced by triptolide. Activation of Nrf2 in renal cells upregulates antioxidant enzymes, reducing ROS levels and preserving mitochondrial integrity. This protection is critical in preventing triptolide-induced nephrotoxicity, as Nrf2-deficient cells show significantly higher levels of apoptosis and mitochondrial dysfunction. Studies suggest that enhancing Nrf2 activity in the kidneys may provide a promising strategy to mitigate triptolide-induced renal damage [63].

Moreover, triptolide’s effects on the gastrointestinal tract and its potential to cause severe gastrointestinal injury through oxidative stress and inflammation further underscore the importance of Nrf2. Activation of Nrf2 in these tissues can attenuate oxidative damage and reduce inflammation, offering protection from triptolide’s adverse effects [136].

In conclusion, the Nrf2 pathway protects against triptolide-induced oxidative damage across various organs. By enhancing antioxidant defenses, Nrf2 activation mitigates triptolide-induced toxicity in the liver, heart, kidneys, and other organs. Continued research into Nrf2 activators may provide novel therapeutic strategies to enhance the safety and efficacy of triptolide, enabling its broader clinical use for treating inflammatory diseases and cancer.

### 8.4. TNF-α

Triptolide reduces both circulating TNF-α levels and the cells’ response stimulated by TNF-α. Triptolide has been shown to inhibit the synthesis and secretion of TNF-α in various models. For instance, Yang and colleagues reported that triptolide effectively reduces the levels of TNF-α and other inflammatory cytokines in a rat model of chronic glaucoma [137]. Additionally, triptolide can suppress TNF-α levels in the serum of a rat model of arthritis, thereby mitigating inflammatory responses [138].

In addition to reducing circulating TNF-α levels, triptolide has been shown to affect the cellular response to TNF-α. For example, triptolide sensitizes breast cancer cells to TNF-α-induced apoptosis by inhibiting the nuclear factor-kappa B NF-κB pathway, which is crucial for TNF-α signaling [130]. Triptolide has also been shown to sensitize acute myeloid leukemia cells to TNF-α Related Apoptosis Inducing Ligand (TRAIL), a different ligand of the TNF- α receptor [18].

Suppression of TNF-α levels is caused by inhibition of NF-κB [130]. Thus, the decrease in circulating TNF-α levels observed after triptolide administration results from triptolide binding to XPB.

### 8.5. miRNA

miRNAs are crucial mediators of triptolide’s therapeutic effects [139]. Triptolide has been reported to induce the expression of specific miRNAs that regulate cellular processes. For instance, Mackenzie et al. demonstrated that triptolide induces the expression of miR-142-3p, which acts as a negative regulator of HSP70 and is associated with reduced pancreatic cancer cell proliferation [140]. Triptolide also modulates miRNAs involved in inflammatory responses. In one study, triptolide inhibited the proliferation of HaCaT cells induced by IL-22 through the upregulation of miR-181b-5p, indicating a role for triptolide in regulating miRNAs associated with inflammation [141].

## 9. Structural Basis of Triptolide Interactions with XPB

**Figure 5:**
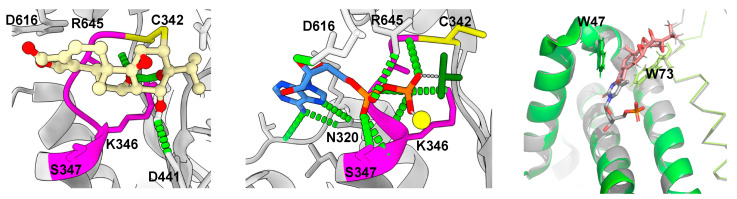
Models of triptolide bound to human XPB and DCTPP1. (**Left**): triptolide (ball and sticks with O atoms in red) covalently docked at residue Cys342 (yellow stick) of human XPB (gray ribbons) (PDB ID: 7NVV); (**middle**): ADP (sticks with N atoms in blue and O atoms in red)-BF3 (green sticks) bound to human XPB (gray ribbons) (PDB ID: 7NVV); (**right**): Superimposition of triptolide (light orange sticks with O atoms in red and H atoms in white) bound to human DCTPP1 (PDB ID: 7MU5) (monomer A in green cartoon while monomer B in lime ribbons) over dCMP (gray sticks with N atoms in blue and O atoms in red) bound to *M. musculus* dCTPase (PDB ID: 6SQW) (monomer A in gray cartoon while monomer B in gray ribbons). Bright green dashed lines indicate H-bond interactions. The Walker A motif of human XPB is highlighted by a magenta color.

The interactions of XPB with triptolide are one of the best characterized among all triptolide targets [15,20,121]. Triptolide irreversibly binds to XPB by a covalent link to the residue Cys342 of XPB through the 12,13-epoxide group [20], resulting in the most potent interactions among all triptolide targets and supporting the antitumor activity of triptolide at low concentrations [47]. When we covalently attach triptolide to the residue Cys342 in the HsXPB structure [122] and perform covalent docking using AutoDock4FR [123], triptolide fits in a pocket partially overlapping with the β- and γ-phosphate groups of ATP bound at the Walker A motif (Figure 5). Many of the same residues are involved in binding triptolide and ATP. Some of the residues implicated by our docking analysis (Lys346, Cys342, Gly343) are all participating in interactions with the ADP bound to XPB (PDB ID: 7NVV). Interestingly, structural analysis on triptolide bound to human DCTPP1 (PDP ID: 7MU5) revealed a much weaker interaction (Figure 5). The basic structural unit of mammalian DCTPP1 is a dimer with two active sites, each formed by residues from two monomers [124]. Comparing the human DCTPP1-triptolide complex structure with the structure of *M. musculus* dCTPase (a DCTPP1 homolog) bound with dCMP (PDB ID: 6SQW), triptolide binds at the active site of DCTPP1 but orients opposite to dCMP (Figure 5) with the ring D of triptolide partially overlapping with the cytosine base of dCMP. Two key tryptophan residues (W47 from monomer A and W73 from monomer B) sandwich triptolide with pi-interactions (Figure 5). These structural results further support that XPB is the primary target for triptolide in antitumor activities.

## 10. Conclusions and Prospective

Here, we reviewed the chemical properties of triptolide and its broad biological activities with severe cytotoxicities and tremendous medical benefits, the Yin and Yang of triptolide applications. We propose that the irreversible covalently binding of triptolide with XPB causes the overall inhibition of the genome transcription and DNA repair, forming the basis for its antitumor and cell death activities. On top of that, triptolide is able to interact with other proteins that are key to some important cellular and signal pathways, showing extremely promising potential as a valuable drug for the treatment of cancer, inflammation, immunosuppression, etc. It is obvious that the mechanisms of triptolide actions are far from clear. Future studies on triptolide/derivatives and target proteins will help to improve its medical efficacy and limit or eliminate its toxicities. As a prominent functional component in TWHF, a traditional Chinese herb, triptolide provides a wonderful example of how to discover valuable drugs from traditional medicine worldwide.

## Figures and Tables

**Table 1:** Summary of Delivery Systems.

Delivery System	Year	Type of Study	Route of Admin	Pros	Ref.
Liposome Hydrogel	2015	in vivo	transdermal	Slower time to peak, more extended stability of plasma concentration vs. oral administration, and improved bioavailability.	[90]
Polymeric Micelles	2008	in vitro and in vivo	intravenous	Prolonged blood circulation, reduced cumulative toxicity, deeper tumor penetration, and improved endocytosis.	[91]
PLGA Nanoparticles	2021	in vitro	intranasal	Novel nasal brain targeting preparation, PLGA is FDA approved, has a sustained release profile, better transcellular permeability, reduced cytotoxicity, and attenuated oxidative stress.	[92]
Stimuli-Responsive Nanoparticles	2021	in vitro and in vivo	intravenous	pH/redox-dual-stimuli-responsive drug release profile, selective tumor uptake and high tumor tissue accumulation, CD44 targeting.	[89]
Exosomes	2019	in vitro and in vivo	intraperitoneal	Antitumor effect in vivo is superior to free triptolide.	[75]
Microemulsions	2008	in vivo	transdermal	Only mild reversible skin irritation signs and a good safety profile were observed.	[93]
Nano-Drugs	2021	in vitro and in vivo	intravenous	Good cancer targeting, enhanced apoptosis, remarkable tumor growth inhibition in vivo, and reduced toxicity.	[74]
Solid Dispersions	2022	in vitro and in vivo	intragastric	Enhanced oral bioavailability and improved antitumor activity.	[77]

**Table 2:** List of proteins directly targeted by triptolide.

Target	Triptolide Binding Mode	Activities	Affected Functions	Validation	Ref.
**XPB**	Irreversible	ATPase	NER, transcription	Immunoprecipitation and immunoblot	[7,16,21,101]
**DNA-PKcs**	Irreversible	Kinase	DNA repair, cell cycle	Computational prediction, thermal shift assay, co-immunoprecipitation, activity assays	[7,101,102,103]
**HSP70**	Reduced Expression	Chaperone	Protein folding, stress response	Immunoblot, qPCR, immunohistochemistry, assays	[14,104,105,106]
**PC-2**	Reversible	Calcium channel	Calcium release	Chromatographic protein fractionation, MALDI-MS analysis, and immunoblot	[7,45,101,107,108]
**ADAM10**	Reduced Expression	Proteinase	Protein metabolism	Affinity chromatography and mass spec	[7,101,109]
**DCTPP1**	Reversible	Pyrophosphatase	Nucleotide metabolism	Photoaffinity pull-down assay	[7,101,110,111]
**TAB1**	Reversible	TAK1 (kinase) activation	TAK1 kinase	Pull-down assays, chemical proteomics	[7,101,112]
**PRXI**	Irreversible	Chaperone	Protein folding	Competition binding assay, size exclusion chromatography, mass spectrometry, activity assays, small molecule probes, immunoblot	[7,101,113]
**Erα**	Reversible	Mitogenic	Proliferation	Computational prediction, surface plasmon resonance, isothermal titration calorimetry, reporter gene assays	[114]

## Abbreviations

Alzheimer’s Disease (AD), A-disintegrin and metalloprotease-10 (ADAM10), Autosomal Dominant Polycystic Kidney Disease (ADPKD), Antioxidant Response Elements (ARE), dCTP pyro-phosphatase 1 (DCTPP1), Diabetic Nephropathy (DN), DNA–PK complex kinase subunit (DNA–PKcs), Glutamate Cysteine Ligase Catalytic Subunit (GCLC), glutathione (GSH), glucose transporters (GLUTs), Heme Oxygenase-1 (HO-1), Heat Shock Protein 70 (HSP70), (5R)-5-hydroxytriptolide (LLDT8), Dmammalian Target Of Rapamycin (mTOR), nucleotide excision repair (NER), Nuclear Factor-kB (NF-κB), NAD(P)H quinone oxidoreductase 1 (NQO1), Nuclear factor erythroid 2-related factor 2 (Nrf2), PolyCystin-1 (PC-1), PolyCystin-2 (PC-2), Poly(lactic-co-glycolic) Acid (PLGA), Peroxiredoxin I (Prx I), Reactive Oxygen Species (ROS), RNA polymerase II (RNAPII), Structure-activity relationship (SAR), Transforming growth factor β-activated kinase 1 (TAK1), TAK1 binding protein (TAB1), Transcription factor IIH (TFIIH), Tumor Necrosis Factor (TNF)-α, Tripterygium wilfordii (TWHF), Xeroderma Pigmentosum B (XPB).
